# A novel European H5N8 influenza A virus has increased virulence in ducks but low zoonotic potential

**DOI:** 10.1038/s41426-018-0130-1

**Published:** 2018-07-19

**Authors:** Christian Grund, Donata Hoffmann, Reiner Ulrich, Mahmoud Naguib, Jan Schinköthe, Bernd Hoffmann, Timm Harder, Sandra Saenger, Katja Zscheppang, Mario Tönnies, Stefan Hippenstiel, Andreas Hocke, Thorsten Wolff, Martin Beer

**Affiliations:** 1 Institute of Diagnostic Virology, Friedrich-Loeffler-Institut, Greifswald-Insel Riems, Germany; 2 Department of Experimental Animal Facilities and Biorisk Management, Friedrich-Loeffler-Institut, Greifswald-Insel Riems, Germany; 3 Unit 17 Influenza and other Respiratory Viruses, Robert Koch Institut, Berlin, Germany; 4 Department of Internal Medicine/Infectious Diseases and Respiratory Medicine, Charité-Universitätsmedizin Berlin, corporate member of Freie Universität Berlin, Humboldt – Universität zu Berlin, and Berlin Institute of Health, Berlin, Germany; 5 HELIOS Clinic Emil von Behring, Department of Thoracic Surgery, Chest Hospital Heckeshorn, Berlin, Germany

## Abstract

We investigated in a unique setup of animal models and a human lung explant culture biological properties, including zoonotic potential, of a representative 2016 highly pathogenic avian influenza virus (HPAIV) H5N8, clade 2.3.4.4 group B (H5N8B), that spread rapidly in a huge and ongoing outbreak series in Europe and caused high mortality in waterfowl and domestic birds. HPAIV H5N8B showed increased virulence with rapid onset of severe disease and mortality in Pekin ducks due to pronounced neuro- and hepatotropism. Cross-species infection was evaluated in mice, ferrets, and in a human lung explant culture model. While the H5N8B isolate was highly virulent for Balb/c mice, virulence and transmissibility were grossly reduced in ferrets, which was mirrored by marginal replication in human lung cultures infected ex vivo. Our data indicate that the 2016 HPAIV H5N8B is avian-adapted with augmented virulence for waterfowl, but has low zoonotic potential. The here tested combination of animal studies with the inoculation of human explants provides a promising future workflow to evaluate zoonotic potential, mammalian replication competence and avian virulence of HPAIV.

## Introduction

In nature, sixteen different hemagglutinin (HA) and nine neuraminidase (NA) subtypes of avian influenza (AI) viruses circulate in aquatic wild birds, and some of them transmit to domestic poultry and occasionally to mammals. During the last decades, a steadily increasing burden of AI viruses (AIV) on poultry production was noticed worldwide. In addition, the sustained circulation in poultry of potentially zoonotic AIV presented global threats for human health^1,2^. Since the first report of highly pathogenic avian influenza (HPAI) H5N1 virus, A/goose/Guangdong/1/96 (gs/GD), in China 1996, descendants of this virus (gs/GD lineage) continue to spread among avian species. The hemagglutinin (HA) gene of these viruses has evolved into ten distinct phylogenetic clades (clades 0–9)^3^. During the evolution of gs/GD-like HPAI H5N1 viruses, frequent reassortment events with other co-circulating AIVs in several countries were noticed, giving rise to different sub- and genotypes^4,5^. Significantly, HPAI H5N1 viruses of the gs/GD-lineage circulating in poultry markets were detected for the first time in 18 human patients in Hong Kong in 1997, of which 6 did not survive the infection. These viruses spread from Asia to European and African countries and also re-emerged in 2003 in humans. According to the World Health Organization (http://www.who.int/influenza/human_animal_interface/en/), HPAIV H5N1 viruses belonging to clades 1 and 2 of the gs/GD lineage have caused 859 human infections and 453 deaths by 25 July 2017^6^, in almost all cases as a result of zoonotic transmissions.

The ancestral strain of HPAIV H5N8 was isolated from ducks in China in 2010 and designated as clade 2.3.4.4 that has evolved into the two clusters H5N8A (Buan/Donglim-like) and H5N8B, first detected in Korean birds (Gochang-like). HPAI H5N8A were reported among wild birds in Asia and regionally spread to and among domestic birds in China, South Korea, and Japan^7,8^. During 2014 and 2015, HPAIV H5N8 clade 2.3.4.4A viruses have caused outbreaks in poultry in Europe, North America, and East Asia^9^.

In spring 2016, a novel reassortant H5N8 virus of clade 2.3.4.4 was identified in wild birds found at the Russian-Mongolian border region associated with mortality in wild birds^10^. In fall of 2016, descendants of this virus were detected in many countries in Europe, Asia, the Middle East, and Africa, and have been assigned to group B within clade 2.3.4.4^10–13^. These novel HPAI H5N8B viruses were not only responsible for substantial economic losses in poultry holdings, but also caused unprecedented mortality in many wild bird species and zoo bird populations throughout Europe and beyond^12^. Primary 2.3.4.4B incursions have been linked to infected migratory wild birds although secondary spread among poultry populations has been reported as well^11,14,15^. In Germany, more than 1200 wild bird cases were reported and in 91 poultry farms a total of 1.2 million birds was killed or sacrificed, and several zoos were affected (www.fli.de). The expanding geographic range of the H5N8B viruses paralleled and even superseded in temporal and geographic terms the rapid spread of viruses of the gs/GD lineage, clade 2.2, in Europe in 2005–2006^12,13,16^. As compared to the spread of the more contemporary H5N8 2.3.4.4 A, the B lineage was much more vigorous in spread and intensity, in geographical and temporal terms and inasmuch as numbers of affected wild birds and species are concerned. From a European perspective a ranking of the severity of gs/GD outbreaks is like this: 2.3.4.4B (2016/17), 2.2 (2005/6), 2.3.2.1c (2010) and 2.3.4.4 A (2014/15)

European clade 2.3.4.4 group B HPAI H5N8 viruses have reassorted to generate a series of different genotypes and subtypes (H5N5, H5N6)^12,15,17^. While HPAI H5N8 viruses of clade 2.3.4.4A were considered to be of low risk of zoonotic transmission^18^, it is possible that the new 2.3.4.4B reassortants may express altered phenotypic characteristics as demonstrated by their unusual virulence for anseriform species in nature. In addition, HPAI H5N6 viruses of clade 2.3.4.4C are known to harbor considerable zoonotic potential as they were detected in at least 12 fatal human cases^19^. Wild waterfowl and domestic ducks were found to be important in the emergence, spread and maintenance of H5N8 HPAI^20^ related to the fact that gs/GD lineage HPAIV induce, in aquatic wild bird species and in domestic ducks, a range of clinical patterns from asymptomatic infections to severe and fatal disease^21,22^.

In the current study, a comparative in vivo and in vitro evaluation provides important data, which allowed a risk evaluation of the novel European HPAIV H5N8 clade 2.3.4.4B featuring substantial evolutionary changes in the HA and internal genes. The selected strains of clade 2.3.4.4A (2014) and B (2016) showed a nucleotide identity of 87% to 96% for the different segments (NA: 96%; HA: 94%; NS: 93%; NP: 93%; M: 92%; PA: 92%; PB1: 91%; PB2: 87%). The pathogenesis and transmissibility of the novel HPAIV H5N8B was extensively studied in the duck model. In order to assess the risk of human infections, its virulence in mammalian species and the potential for zoonotic transmission was investigated in mice (*Mus musculus*, Balb/c) and ferret (*Mustela putorius furo*) animal models, as well as in explanted human lung cultures infected ex vivo.

## Results

### Permissiveness, pathogenesis, and transmissibility of European HPAIV H5N8 reassortants in ducks and chickens

We compared in a first step recent H5N8 virus isolates collected during the epizootics in Germany in 2014 and 2016 (DE14-H5N8A and DE16-H5N8B), whose HA genes represented groups 2.3.4.4A and B, respectively, in chicken. After intravenous infection with DE14-H5N8A and DE16-H5N8B, all 10 inoculated chickens died within 2 days, resulting in an intravenous pathogenicity index (IVPI) of 2.81 and 2.93, respectively^12^. Thus, by legal terms, both viruses were highly pathogenic in chickens without marked differences in virulence and induced clinical signs. Also by contact with the i.v. inoculated chickens, the mortality of sentinel chicken reached 100% for both viruses.

**Fig. 1:**
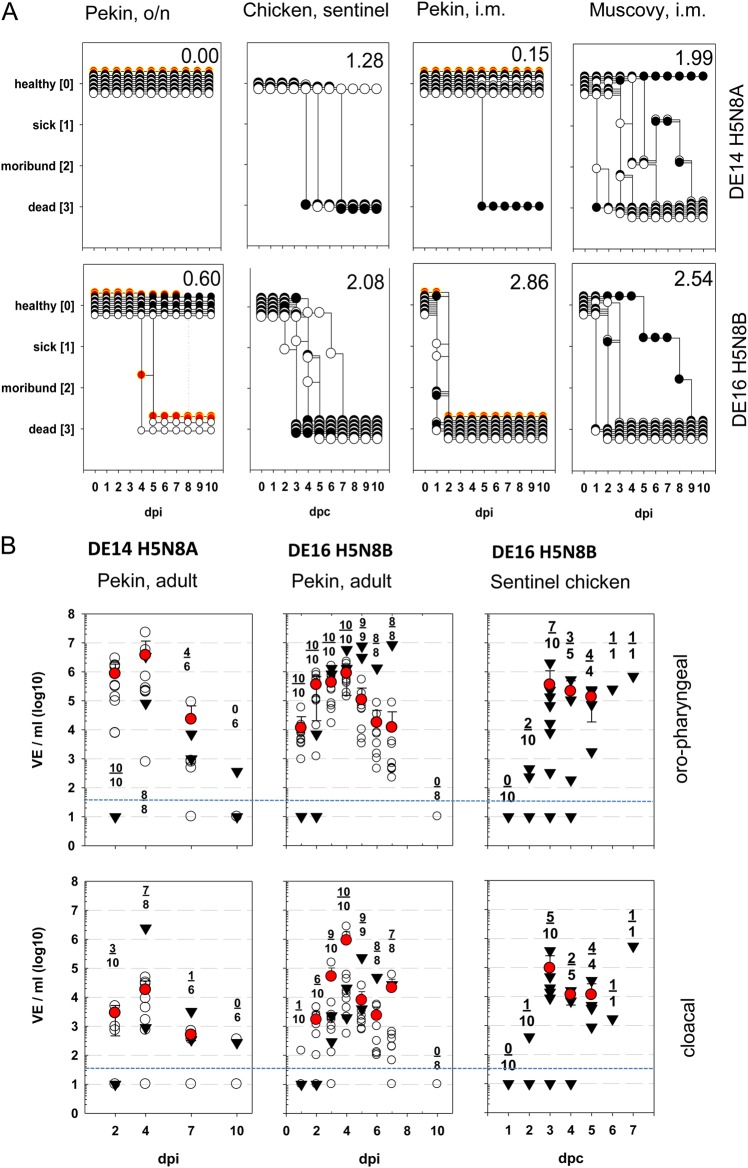
Clinical course and viral shedding of HPAIV H5N8-infected ducks and chicken. **A** Depicted is the clinical course of ducks during acute phase after infection with HPAIV H5N8 viruses belonging either to clade 2.3.4.4A (DE14-H5N8A) (upper panel) or clade 2.3.4.4B (DE16-H5N8B) (lower panel). Adult pekin ducks were inoculated oronasally (o.n.) (left panel) and 1-week old pekin- or muskovy ducks were inoculated intramuscularly (i.m.). In addition, clinical course of sentinel ducks (red symbols) and sentinel chickens (graphs on the right) kept in direct contact to adult ducks is shown. Numbers within the figures give the clinical score for individual groups according to the IVPI scoring system^59^. **B** Oro-pharyngeal (upper panel) and cloacal swabs (lower panel) were taken from adult ducks and chickens (sentinel to DE16 H5N8B infected ducks) at indicated days post infection (dpi) or days post contact (dpc) and tested by RT-qPCR. Individual results of detected RNA copy numbers are given as virus equivalents (VE), calculated by using a set of standards applied in each run, with the blue line indicating the threshold. Besides individual results of inoculated birds (open circles), arithmetic mean and s.d. of infected virus-positive samples (red dots) are shown, as well as sentinel birds (triangles). Numbers within the figures provide the number of virus positive -and the number of total tested infected animals at each time point

Oculo-nasal infection of adult Pekin ducks and geese with HPAIV DE14-H5N8A did not induce any clinical signs (Fig. 1A). In contrast, application of DE16-H5N8B caused death of 2 out of 10 inoculated adult ducks within 4 and 5 days post infection (dpi), resulting in a clinical score of 0.6 (Fig. 1A). Interestingly, also the two sentinel ducks of the H5N8B trial co-housed with the infected animals died on day 4 and day 8, respectively, i.e., 3 and 7 days after having contact to inoculated animals. These differences in the pathogenicity between DE14-H5N8A and DE16-H5N8B became more pronounced following i.m. inoculation in 10 1-week-old Pekin ducks. Whereas only one of the DE14-H5N8A inoculated ducklings died at 3 dpi, all 10 ducklings inoculated with DE16-H5N8B succumbed to infection by 2 dpi, including 2 sentinel animals (Fig. 1A, *p* = 0.0003; compare Pekin, i.m. upper panel to Pekin, i.m. lower panel). The clinical course in Muscovy ducklings after i.m. inoculation of DE14-H5N8A was much more severe compared to Pekin ducklings (Fig. 1A, *p* = 0.0007; compare Pekin, i.m. upper panel to Muscovy, i.m. upper panel). Interestingly, progressive disorders of the central nervous system like tremor and opisthotonus prevailed in Muscovy ducks with a delayed onset of mortality after infection with H5N8B.

At the indicated times, swab samples were taken from the oro-pharynx and separately from the cloaca of individual birds and tested for viral excretion. Despite a healthy appearance of adult ducks and geese after infection with DE14-H5N8A, both species shed virus already 2 dpi: pharyngeal excretion was more pronounced compared to cloacal shedding (Fig. 1B; compare upper panel to lower panel). Viral load in the pharyngeal swab samples stayed high on 4 dpi, with a slight increase in viral genome load from 8.5 × 10^5^ VE/ml to 3.7 × 10^6^ VE/ml for ducks, and from 1.6 × 10^6^ VE/ml to 3.1 × 10^6^ VE/ml for geese (Supplementary Fig. S1). At that time also 7 from 8 cloacal swab samples were AIV-positive. On 7 dpi viral titers declined, but still 4 out of 6 duck samples were AIV-positive, and shedding persisted until 14 dpi for individual birds, with minute amounts of viral RNA detectable in pharyngeal (geese 14 dpi *n* = 3, see Supplementary Fig. S1; duck *n* = 1, data not shown) and cloacal swabs (geese 14 dpi *n* = 1, see Supplementary Fig. S1) of geese and/or ducks, respectively. Virus was transmitted efficiently to sentinel ducks and geese, with a similar course of infection as seen in the inoculated birds. In addition, 3 out of 4 sentinel chickens became infected and died within 7 days of contact (4, 5 and 7 days post contact (dpc)).

Remarkable, however, is the observation that on 14 dpi both sentinel geese still yielded viral RNA in pharyngeal swab samples, indicating virus replication and possibly shedding for up to 2 weeks.

Following infection with DE16-H5N8B virus, viral shedding started at 1 dpi, readily detectable in pharyngeal swabs from all 10 oronasally (o.n.) inoculated adult ducks, but only in one cloacal swab sample. By 2 and 3 dpi, cloacal swab samples from 6 and 9 ducks, respectively, yielded virus RNA, reaching the peak of shedding on 4 dpi with 8.7 × 10^5^ VE/ml and 4.6 × 10^5^ VE/ml for pharyngeal and cloacal swabs, respectively (Fig. 1B). Viral loads in swab samples declined, by 7 dpi, all animals alive shed virus by pharyngeal and cloacal routes. Thereafter, none of the samples tested positive. In line with the shedding data was the finding that DE16-H5N8B virus was transmitted efficiently to sentinel ducks (Fig. 1B, triangles) and to 10 sentinel chickens, that died within 7 days after contact (Fig. 1A). In line with the high level of virus replication, antibody responses developed fast in both waterfowl species and were already detectable on 7 dpi (Supplementary Fig. S2).

### Pathology of infected poultry—pronounced neuro- and hepatotropism of DE16-H5N8B in ducks

None of the ducks and geese infected with DE14-H5N8A had any pathological changes when euthanized 2 and 4 dpi. However, viral RNA was readily detected in almost all organs regardless whether animals were sacrificed on 2 or 4 dpi (Supplementary Fig. S3A) with lowest viral loads in the brain. Analyses of tissue from ducks and geese taken at the end of the experiment, i.e., 21 dpi exhibited only very mild alterations, mainly evident as focal, acute, necrotizing hepatitis with a low degree of lymphohistiocytic infiltration in the periportal fields. For all animals, a lymphatic depletion affecting the spleen and caecal tonsils was present. No lesions were present in the brain of ducks and geese after DE14-H5N8A infection. This observation contrasts findings in the sentinel chicken in which virus loads exceeded the levels found in waterfowl and were detected in all organs including the brain. These findings coincided with antigen detection by immunohistochemistry (IHC) in the different tissues. In all animals, the infraorbital sinus proved to be a site of fulminant virus replication with heavy antigen staining. Strikingly, no AIV-antigen was detected in the brain of waterfowl (Table 1 DE14-H5N8A). Histopathologic alterations in chicken infected with DE14-H5N8A were dominated by necrosis and/or necrotizing inflammation in multiple organs including polioencephalitis and are in agreement with previous reports for HPAIV infections^21,23^.

**Table 1:** Semiquantitative AIV antigen detection in avian species by IHC

	DE14-H5N8A									DE16-H5N8B				
	Geese				Ducks				Chickens	Ducks				Chickens
Animal ID^a^	G25	G23	G26	G21	E15	E16	E20	E17	n = 3	E38	E39	E49	E35	n = 10
Time (dpi)	2		4		2		4		4–7	4	5	5	8	3–7
Sinus	+++	++	++	++	++	++	++	++	+-+++	++	**n.d**.	++	+	++-+++
Lung	Neg	Neg	Neg	Neg	Neg	++	Neg	+	+	+	++	++	+	++-+++
**Brain**	Neg	Neg	Neg	Neg	Neg	Neg	Neg	Neg	++-+++	+	+	++	++	++-+++
Proventriculus	Neg	+	+	+	Neg	+	Neg	Neg	++	+	+	Neg	Neg	++-+++
Duodenum	Neg	Neg	Neg	Neg	Neg	Neg	Neg	Neg	Neg	+	++	+	+	++
Pancreas	Neg	Neg	+	+++	Neg	Neg	Neg	Neg	+++	++	++	++	**n.d**.	+-++
**Liver**	Neg	+	Neg	++	Neg	Neg	Neg	Neg	Neg—+	+++	+++	+++	+	+-++
Kidney	++	+	Neg	+	Neg	Neg	Neg	Neg	Neg—+	Neg	+	Neg	Neg	++-+++
Heart	Neg	Neg	Neg	Neg	Neg	Neg	Neg	Neg	++	++	++	+	+	++-+++
Ovary/Testis	Neg	Neg	Neg	+	Neg	Neg	Neg	Neg	++	+	++	+	Neg	++-+++
Adrenal gland	Neg	Neg	Neg	Neg	Neg	Neg	Neg	Neg	Neg	+++	++	++	+	++-+++
Spleen	Neg	+	Neg	Neg	Neg	Neg	Neg	Neg	Neg -++	+	+	+	+	++-+++

*n.d.*/ not done

^a^Individual animal specific number tag

In marked contrast, DE16-H5N8B infected adult Pekin ducks revealed gross lesions dominated by severe, acute, diffuse, necrotizing hepatitis, mild to moderate, acute, multifocal petechiae as well as moderate to severe, acute, diffuse congestive pulmonic hyperemia. Viral loads were prominent in the lungs (2.3 × 10^5^‒3.6 × 10^7^ VE/ml). However, in contrast to DE14-H5N8A infected ducks, high abundance of viral RNA in the brain of all 4 dead ducks (Supplementary Fig. S3B; 1.4 × 10^7^‒5.4 × 10^6^ VE/ml) was noted, reaching levels similar to infected sentinel chicken (Supplementary Fig. S3C; 3.8 × 10^6^‒2.0 × 10^9^ VE/ml). The highest viral loads were observed in the liver of DE16-H5N8B-infected ducks with gross lesions for animals that died 4 and 5 dpi (3.5 × 10^8^‒1.5 × 10^9^ VE/ml). These values even exceeded the viral load found in livers of sentinel chickens (1.5 × 10^5^‒7.8 × 10^6^ VE/ml). Interestingly, in the duck that died late, i.e., 8 dpi, viral load in the liver was lower (1.5 × 10^4^ VE/ml). The virological observations coincide with the pattern of antigen distribution as detected by IHC  in the tissues of the ducks (Table 1).

**Fig. 2:**
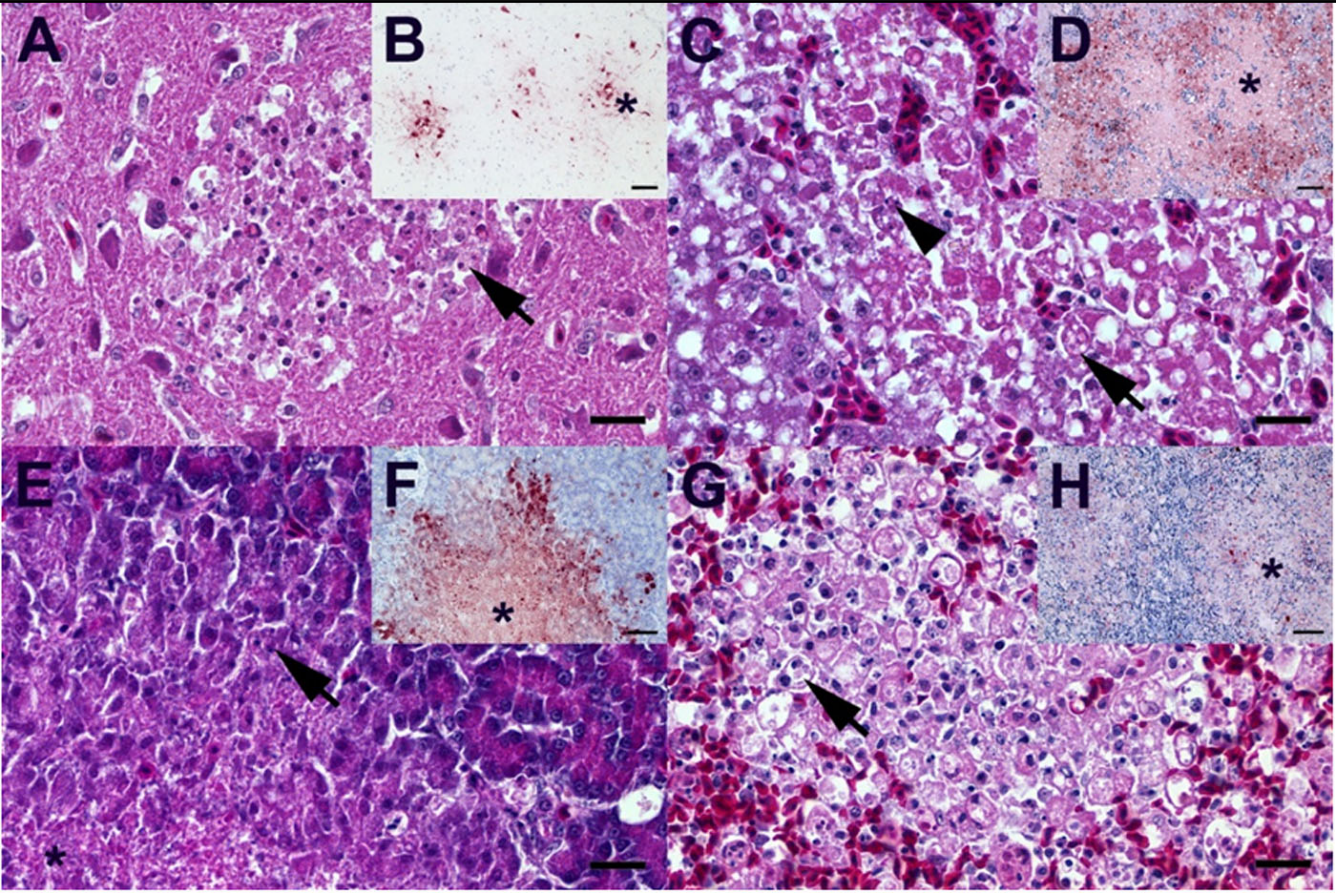
Characteristic light microscopic findings in ducks infected with DE16-H5N8B. **A** Duck, 8 dpi, brain. Moderate, subacute necrotizing polioencephalitis with infiltration of phagocytic microglia (arrow). **B**  Duck, 8 dpi, brain. Multiple foci (star) of influenza A nucleoprotein-immunoreactive neuronal and glia cells. **C**  Duck, 5 dpi, liver. Severe, acute, hepatic necrosis (necrotizing hepatitis) characterized by cytoplasmic hypereosinophilia and vacuolation (arrow), membraneous rupture and nuclear pyknosis, karyorrhexis (arrowhead) and loss. **D** Duck, 5 dpi, liver. Influenza A virus-nucleoprotein-immunoreactive hepatocytes are typically located at the border of the coalescing necrotizing lesions, whereas only faintly immunoreactive cellular debris is present in the lesion centers (star). **E** Duck, 5 dpi, pancreas. Moderate, acute pancreatic liquefactive necrosis (necrotizing pancreatitis) characterized by granular eosinophilic debris (star) and scant nuclear pyknosis, karyorrhexis (arrow) and loss. **F** Duck, 5 dpi, pancreas. Influenza A virus-nucleoprotein-immunoreactive exocrine pancreatocytes are typically located at the border of the multifocal necrotizing lesions, whereas only faintly immunoreactive cellular debris is present in the lesion centers (star). **G** Duck, 4 dpi, spleen. Severe, diffuse, lymphatic depletion and marked tingible body macrophage (arrow) hyperplasia within the white pulp surrounded by hyperemic red pulp. **H** Duck, 4 dpi, spleen. Oligo- to multifocal discrete influenza A virus-nucleoprotein-immunoreactive cells interpreted as macrophages/dendritic cells, endothelia and faintly immunoreactive debris within the depleted white pulp (star) and less frequently in the hyperemic red pulp. **A**, **C**, **E**, **G** Hematoxylin eosin, bar = 20 µm. **B**, **D**, **F**, **H** Influenza A virus-nucleoprotein immunohistochemistry, avidin-biotin-peroxidase complex method with a polyclonal rabbit anti- influenza A FPV/Rostock/34-virus-nucleoprotein antiserum (diluted 1:750)^65^ 3-amino-9-ethyl-carbazol as chromogen and hematoxylin counterstain, bar = 50 µm

Histopathology of DE16-H5N8B-infected birds revealed necrosis and/or necrotizing inflammation affecting multiple organs with a species-specific degree of severity (Fig. 2 and Supplementary Fig. S4). A significantly higher grade of severe, acute, coalescing to diffuse necrotizing hepatitis was observed in 3 out of four ducks (Fig. 2C), as compared to a mild, oligo- to multifocal, acute, necrotizing hepatitis in 6 out of 9 of the chicken (Supplementary Fig. S4C) (Mann–Whitney *U* test; *p* = 0.034). Immunohistochemistry revealed, that  3 out of 4 DE16-H5N8B-infected ducks displayed a coalescing influenza virus antigen distribution mainly within hepatocytes bordering the coalescing necrotic lesions as well as cellular debris within the lesion centres (Fig. 2D). In agreement with high viral loads in the brain, moderate, multifocal, acute to subacute, necrotizing polioencephalitis with a variable degree of microgliosis was a characteristic finding in all DE16-H5N8B-infected ducks (Fig. 2A), and chickens (Supplementary Fig. S4A). Furthermore, a significantly higher grade of moderate, multifocal, acute, necrotizing pancreatitis was observed in 100% of the ducks (Fig. 2E), whereas only a mild, oligo- to multifocal, acute, necrotizing pancreatitis was present in 70% of the chicken (Supplementary Fig. S4E) (Mann–Whitney *U* test; *p* = 0.028). A moderate to severe, multifocal to diffuse, lymphatic apoptosis, necrosis and/or depletion with variable tingible body macrophage hyperplasia affecting the spleen (Fig. 2G; Supplementary Fig. S4G), multiple mucosa-associated lymphoid tissues, caecal tonsil, bursa and thymus was another important lesion observed in all ducks and chicken. Notably, the duck that was found dead 8 dpi, possibly represents a biological outlier with severe, fibrinonecrotizing pneumonia with vasculitis, thrombosis and hematogenous spread of fungal hyphae most likely due to secondary mycotic infection fostered by HPAI.

Taken together, the clinical correlate of the increased virulence for ducks of DE16-H5N8B compared to DE14-H5N8A is the pronounced neuro- and hepatotropism inducing necrotizing and inflammatory processes in these organs. Furthermore, the striking lack of lymphohistiocytic perivascular infiltrations in these lesions suggests a direct or indirect lymphocytotoxic effect of DE16-H5N8B which could subsequently result in immunosuppression.

As there are considerable published data available with regard to the pathogenicity of H5N8 clade 2.3.4.4 group A viruses in both the mouse and ferret models (*inter alia*^18,24–26^), these inoculation experiments using DE14-H5N8A were not reproduced in accordance with efforts to reduce animal experiments (3 R concept).

### Virus pathogenicity and tissue tropism in mice—H5N8B is virulent for Balb/C mice

**Fig. 3:**
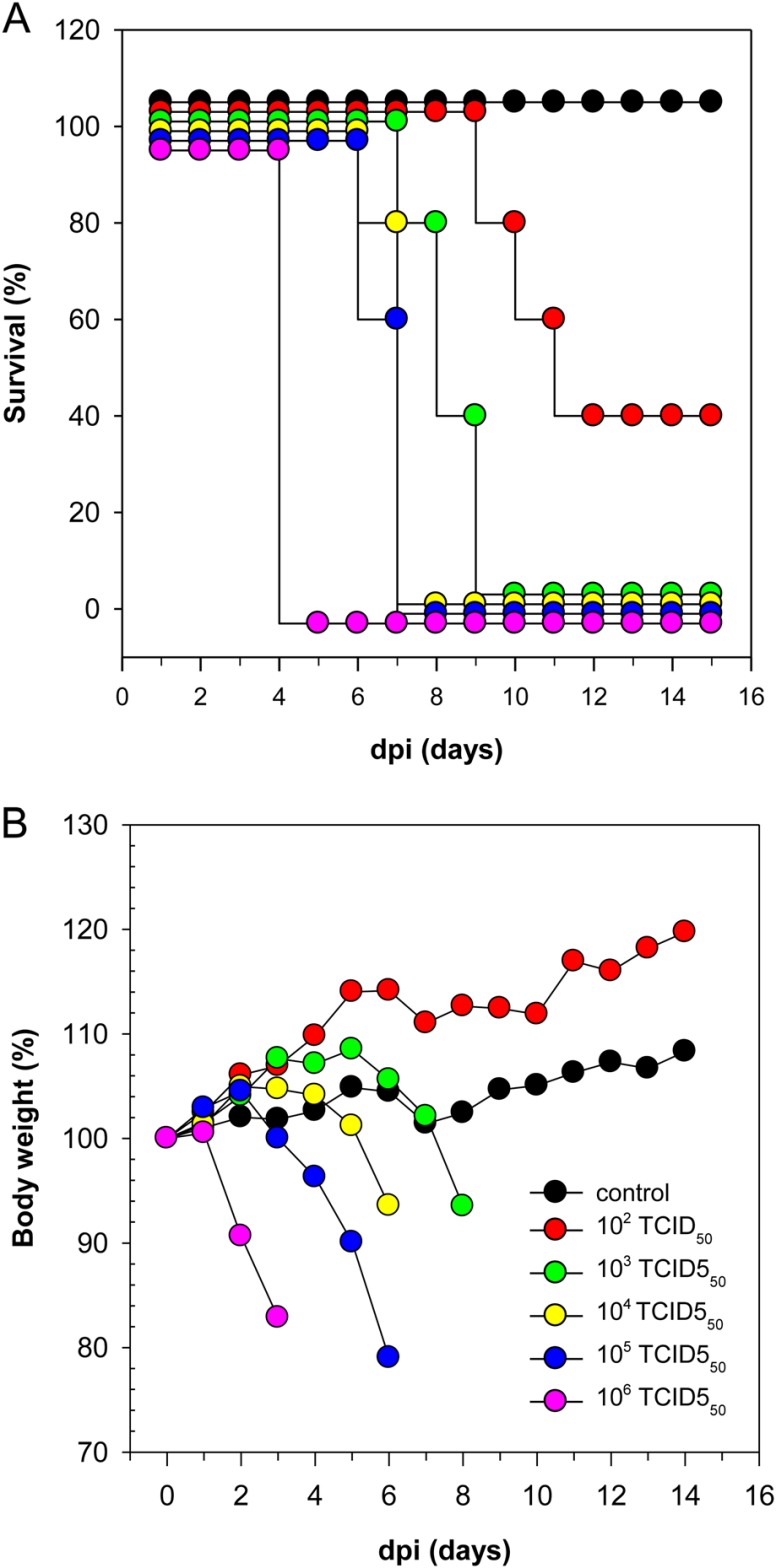
Mean lethal dose 50 (MLD50) titration and clinical course  of DE16-H5N8B in mice. **A** Survival percentage and **B** change in body weight of 4-week old BALB/c mice infected intranasally with five different infectious doses: 10^2^ to 10^6^ TCID50 given in relation to the day post inoculation (dpi). Values generated from control animals are depicted in black, dosage group 10^2^ TCID50 in red, 10^3^ TCID50 in green, 10^4^ TCID50 in yellow, 10^5^ TCID50 in blue, 10^6^ TCID50 in purple

Mice are a mammalian model for pathogenicity studies of HPAI H5 viruses^27^. Here, groups of BALB/c mice were inoculated intranasally with serial 10-fold dilutions of H5N8B. Mice infected with 10^2^ TCID_50_ showed no loss in body weight below the starting weight, while other groups experienced a pronounced decrease in body weight (Fig. 3B). An infectious dose of 10^2^ TCID_50_/animal resulted in a survival rate of only 40%, while all animals receiving higher dosages up to 10^6^ TCID_50_ succumb to the disease or had to be killed by 9 dpi (Fig. 3A). Substantial virus titers in the lungs were found at 3 dpi (individuals exhibiting up to 10^5.6^ TCID_50_/lung sample) and systemic spread of infectious virus to the brain was verified from mice euthanized 6 dpi (data not shown).

**Fig. 4:**
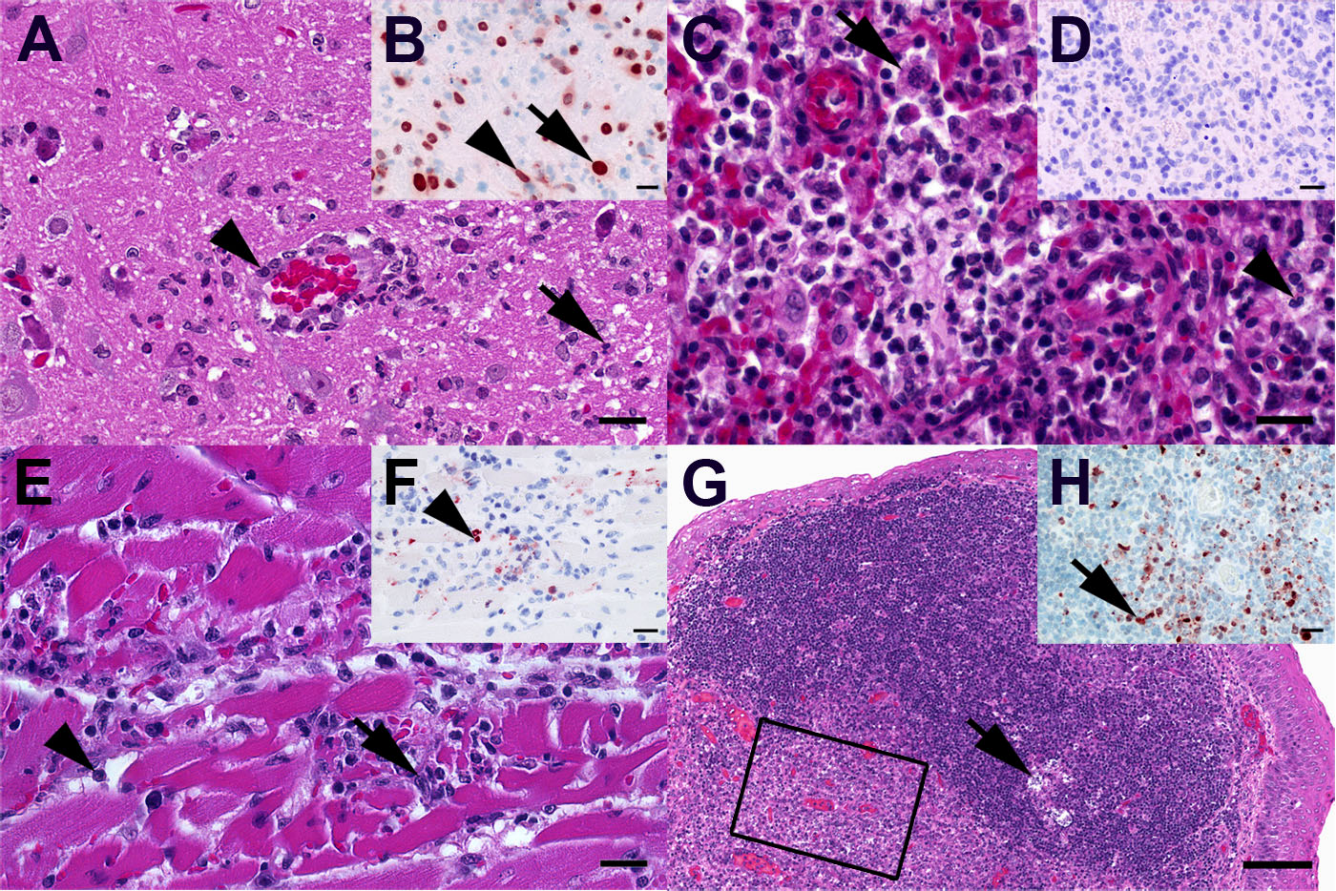
Characteristic light microscopic findings in mice and ferrets infected with DE16-H5N8B. **A** Mouse, medulla oblongata. Moderate, acute, multifocal, necrotizing encephalitis with few neutrophils (arrow), microgliosis and activated hypertrophic vascular endothelium (arrowhead). **B** Mouse, medulla oblongata. Multifocal influenza A nucleoprotein in neurons (arrow) and glial cells (arrowhead). **C** Mouse, lung. Moderate, subacute, multifocal, lymphohistiocytic, interstitial pneumonia with alveolar histiocytosis (arrow), and variable amounts of intraalveolar neutrophils (arrowhead), and alveolar edema. **D** Mouse, lung. No influenza A virus-nucleoprotein-immunoreactive cells in lung section. **E** Mouse, heart. Mild, acute, multifocal, necrotizing myocarditis with infiltrating macrophages (arrow) and neutrophils (arrowhead). **F** Mouse, heart. Oligofocal influenza A virus-nucleoprotein-positive round cells (arrowhead) and cellular debris. **G** Ferret, tonsilla palatina. Prominent follicular germ center with tingible body macrophages (arrow) and an excentric, polar zone of differentiated lymphocytes **H** Ferret, tonsilla palatina. Boxed area in (**G**) Oligofocal round cells (macrophages) with influenza A virus nucleoprotein-positive nuclei (arrow) mainly in interfollicular areas. **A**, **C**, **E**, Hematoxylin eosin, bar = 20 µm. **G** Hematoxylin eosin, bar = 100 µm. **B**, **D**, **F**, **H** Influenza A virus-nucleoprotein immunohistochemistry, avidin-biotin-peroxidase complex method with with a polyclonal rabbit anti- influenza A FPV/Rostock/34-virus-nucleoprotein antiserum (diluted 1:750)^65^ 3-amino-9-ethyl-carbazol as chromogen and hematoxylin counterstain, bar = 20 µm

Histopathology of mice infected with a low dose of DE16-H5N8B (evaluation of dosage group 10^2^ TCID_50_) revealed a mild to moderate, acute, multifocal, necrotizing encephalitis with few neutrophils, microgliosis, degenerating neurons and activated hypertrophied vascular endothelium. Lesions were most prominent in the medulla oblongata but also variably present in other areas of the brain in 4 out of 5 of the DE16-H5N8-infected mice (Fig. 4A, Supplementary Table S1). In the three most severely affected mice, IHC revealed multifocal to diffuse influenza A nucleoprotein in neurons and glial cells of the medulla oblongata (Fig. 4B), whereas only scattered oligofocal immunoreactive cells were detected in other parts of the brain. The lungs of 3 out of 5 of the H5N8-infected mice displayed a moderate, subacute, multifocal, lymphohistiocytic, (bronchiolo-)interstitial pneumonia with alveolar histiocytosis, variable amounts of intraalveolar neutrophils, alveolar edema (Fig. 4C) and intrabronchial cellular debris, but no immunoreactive cells (Fig. 4D). One of the H5N8-infected mice showed a mild, acute, multifocal, necrotizing myocarditis with histiocytic and neutrophilic infiltration (Fig. 4E), and immunohistochemistry demonstrated oligofocal influenza A virus-nucleoprotein-positive cardiomyocytes, round cells and cellular debris within these lesions (Fig. 4F). No obvious lesions and no influenza A virus-nucleoprotein-positive cells were detected in liver, spleen and pancreas of the H5N8-infected mice and in none of the organs of control mice.

Taken together, HPAIV DE16-H5N8B has the ability to replicate in mice without prior adaptation and generally demonstrated a high-pathogenicity phenotype. The final MLD_50_ of DE16-H5N8B in BALB/c mice was calculated as 2.4 × 10^2^/30 µL inoculum.

### Virus pathogenicity in ferrets—low virulence and lack of transmission for HPAIV DE16-H5N8B

**Fig. 5:**
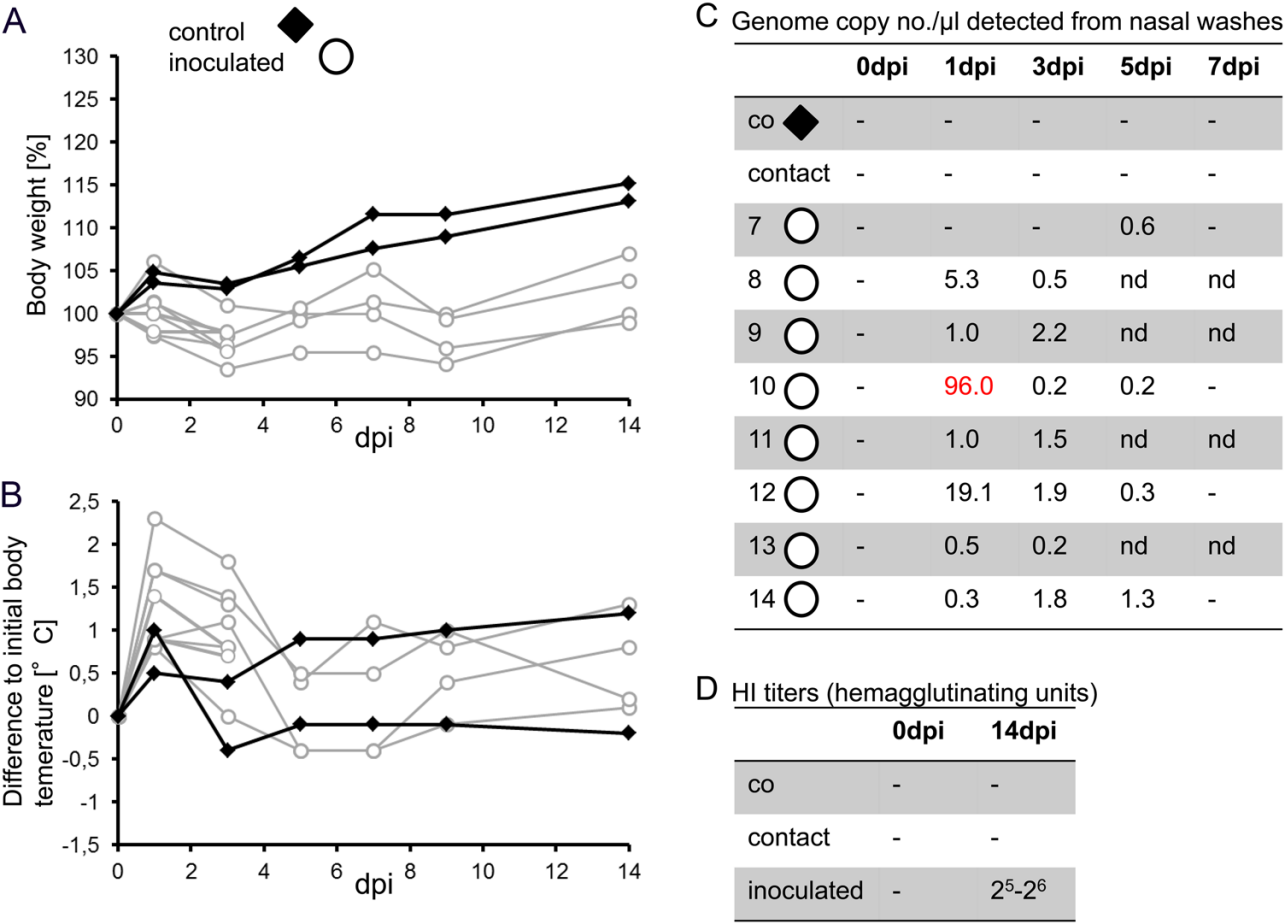
Body weight, temperature, shedding and seroconversion in ferrets. **A** Change in body weight data of inoculated ferrets (open circle) and contact ferrets (filled diamond), and **B** change in body temperature of intranasally infected and contact ferrets. **C** Tabular summary of viral RNA loads detected from nasal washing samples as expressed by genome copy number per µl; the only sample from which virus could be re-isolated is highlighted in red. **D** Tabular summary of hemagglutination inhibition titers in sera from infected and sentinel animals

Ferrets have been extensively used as animal model for zoonotic influenza viruses as they replicate many aspects of human disease^27–30^. In this study, we inoculated ferrets by the intranasal route using 10^6^ TCID_50_ and co-housed them with non-infected sentinel animals to monitor direct transmission. Interestingly, no respiratory symptoms and only minor changes in body weight and body temperature were recorded (Fig. 5). Viral RNA was detected in nasal washes of all inoculated ferrets. However, virus could only be re-isolated from one nasal wash sample on day 1 and moderate viral titers determined from various organ samples (3 dpi). No virus excretion or seroconversion was detected in any of the naive sentinel ferrets, while inoculated animals exhibited NP-specific antibodies in ELISA and HI titers of 2^6^ and 2^7^, respectively, against the homologous antigen (Fig. 5). Hence, no transmission of HPAIV DE16-H5N8B occurred by direct contact/co-housing with infected ferrets.

Histopathology revealed moderate, subacute, oligofocal, lymphohistioplasmacytic rhinitis with focal flattening of the epithelium and loss of cilia in 2 out of 4 of the DE16-H5N8B-infected ferrets sacrificed on 3 dpi (table S1). Mild, multifocal, bronchial and/or alveolar accumulation of eosinophilic, frothy fluid (alveolar edema) but no pneumonia was present in 3 out of 5 infected ferrets. No influenza A virus-nucleoprotein-immunoreactive cells were detectable in the nasal mucosa, trachea and lungs. The palatine tonsil was assessed in two of the infected ferrets, and one out of these displayed prominent follicular germ cells with tingible body macrophages and an excentric, polar zone of differentiated lymphocytes (moderate follicular hyperplasia, Fig. 4G), and oligofocal round cells (macrophages) with influenza A virus nucleoprotein-positive nuclei mainly in the interfollicular areas (Fig. 4H). All ferrets exhibited splenomegaly due to red pulp hyperplasia and extramedullary hematopoiesis, which was interpreted as a species related background change. No other obvious lesions and no influenza A virus-nucleoprotein-positive cells were detected in brain, trachea, lungs, heart, liver, pancreas, kidney, duodenum, jejunum, colon, and skeletal muscle of the DE16-H5N8B-infected ferrets and in none of the organs of a non-infected archival control ferret.

### Poor replication of DE16-H5N8B in human lung culture

**Fig. 6:**
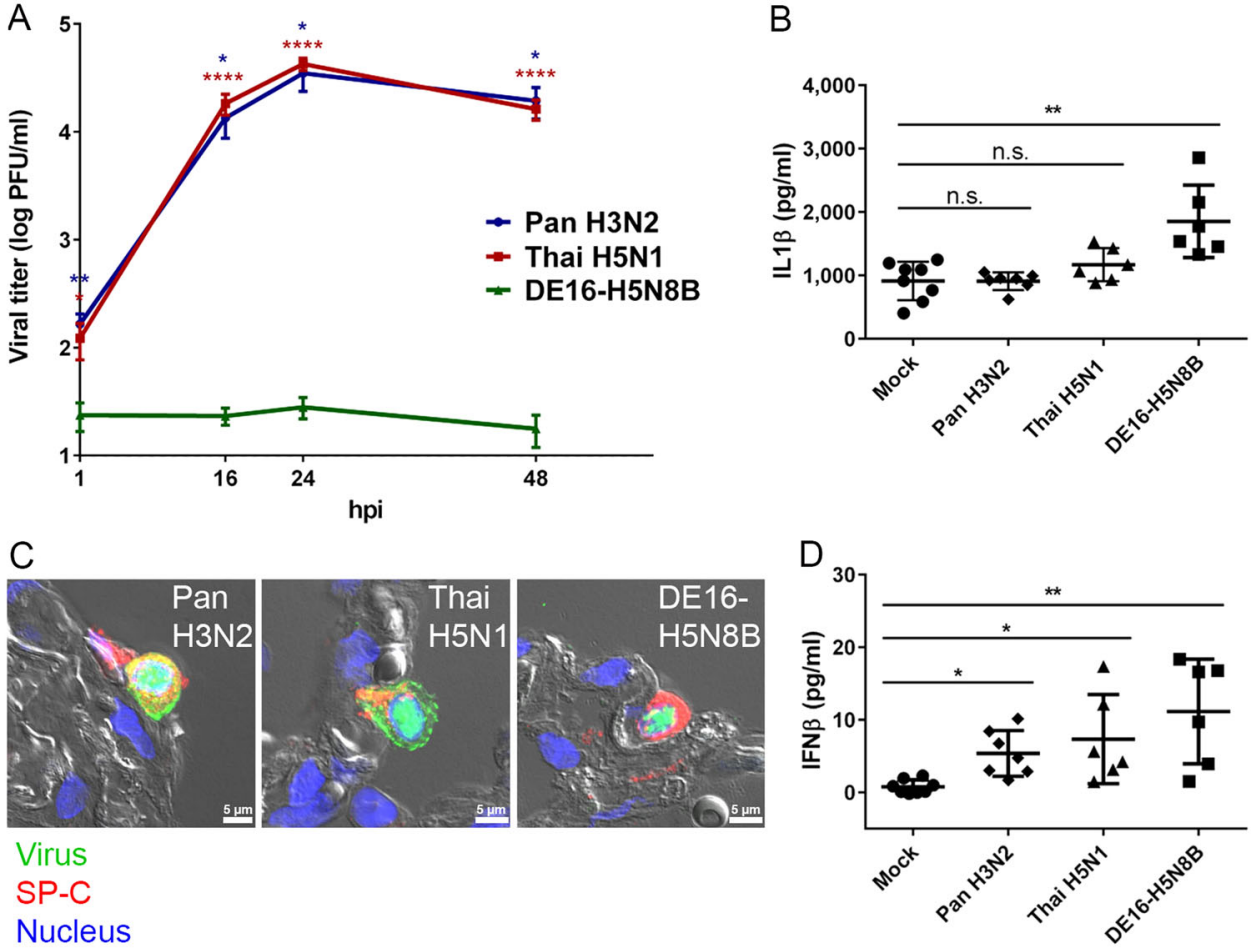
Replication deficiency, increased cytokine induction and cell tropism of DE16-H5N8B in human lung tissue. **A** Tumor-free human lung tissue was infected with 2 × 10^5^ PFU of IAV Pan H3N2, Thai H5N1 or DE16-H5N8B. At 1, 16, 24 and 48 hpi, supernatants of infected lung tissue were harvested and viral titers were determined by standard plaque titration assay. Mean values and standard errors of the means (SEM) from four independent experiments, each done in triplicate, are shown. Asterisks indicate significant differences between IAV strains Pan H3N2 (blue) or Thai H5N1 (red) compared to DE16-H5N8B at each indicated time point (Mann–Whitney *U* test; *p ≤ 0.05; **p ≤ 0.01; ****p ≤ 0.0001). **B**, **D** Tumor-free human lung tissue was infected with 1 × 10^6^ PFU of IAV Pan H3N2, Thai H5N1 or DE16-H5N8B. Aliquots of infected lung culture supernatants 24 hpi were analyzed for the concentrations of IL1β (**B**) or IFNβ (**D**) in pg/ml by commercially available ELISA sets. Data points from three independent experiments done in biological duplicates are shown individually with mean values and standard deviation, respectively. Significance values between virus and mock infected samples are indicated by asterisks (one-way-Anova with Dunn’s multiple comparisons test; *p ≤ 0.05; **p ≤ 0.01). **C** Tumor-free human lung tissue was infected with 1 × 10^6^ PFU of IAV Pan H3N2, Thai H5N1 or DE16-H5N8B. Virus (green) was typically detected in alveolar epithelial type II cells indicated by pro-SP-C (red). Lung structure was visualized by differential interference contrast (grey) and nuclei were counterstained using DAPI (blue). Scale bar 5 µm (panel C)

In order to assess the capacity of DE16-H5N8B to replicate in the lower human respiratory tract, explanted human lung tissue was infected ex vivo with DE16-H5N8B, and in parallel control cultures with seasonal IAV (Pan H3N2) and a human origin isolate of HPAIV H5N1 clade 1 virus (Thai H5N1). Human Pan H3N2 and zoonotic Thai H5N1 both replicated efficiently, whereas the avian DE16-H5N8B hardly propagated in the human lung explants (Fig. 6A). Interestingly, human Pan H3N2 and Thai H5N1 viruses induced lower levels of pro-inflammatory IL1beta and the antiviral IFN-beta compared to DE16-H5N8B (Fig.6B, D). These differences cannot be attributed to an altered cellular tropism, since all three viruses were primarily detected in human alveolar type II pneumocytes as judged by co-staining with influenza virion-antiserum detecting primarily viral NP and M1, and antiserum to pro-SP-C, a type II pneumocyte-specific marker and immunofluorescence microscopy (Fig. 6C). These findings suggest that the DE16-H5N8B virus lacks adaptation for efficient replication in human lung cells.

## Discussion

Comparison of the pathogenesis, and transmissibility of H5N8 viruses of clade 2.3.4.4 groups A and B in chickens and waterfowl, as well as analyses of H5N8 clade 2.3.4.4 group B virus in mice, ferrets, and human lung explant tissue cultures revealed strikingly different, species-specific patterns in avian species and suggests a low zoonotic potential of DE16-H5N8B.

### Experimental infection of poultry–high pathogenicity of HPAIV DE16-H5N8B in ducks

Studies on recent Asian H5 subtype viruses from the Gs/GD lineage (clade 2.3.4.4A) revealed a range of pathobiological outcomes in experimentally inoculated wild and domestic ducks with mortality rates varying from 0 to 20%^18,31–38^. The available data demonstrated the importance of further studies with the recent European HPAIV H5N8 viruses of clades 2.3.4.4A and -B in direct comparison of the strains with different bird species.

Our data clearly demonstrated ducks and geese suffered from a systemic DE14-H5N8A infection with viral spread to several organs. The replication kinetics of DE16-H5N8B in ducks resembled the one of DE14-H5N8A. Compared to chickens, however, the antigen load in waterfowl infected with the clade 2.3.4.4 group A virus (DE14-H5N8A) was strikingly lower. In contrast, in ducks dying between 4 and 5 dpi with the clade 2.3.4.4 B virus (DE16-H5N8B), RNA levels in the organs were similar to that of the sentinel chickens, and in case of the liver were even exceeding the levels detected in chickens. An additional striking difference between the 2014 and 2016 viruses was the neurovirulence. Ducks dying after DE16-H5N8B infection consistently exhibited moderate, multifocal, acute to subacute, necrotizing polioencephalitis with intralesional antigen-positive neuroglial cells and RNA levels in the brain comparable to those of lung samples of the same animals. In contrast, no inflammatory changes and no antigen was detectable in the brains of DE14-H5N8A infected ducks.

This change in pathological manifestation coincided with the clinical outcome: After oculo-nasal infection with DE14-H5N8A virus, all 10 experimental infected adult geese and Pekin ducks as well as the sentinel animals stayed healthy over the entire observation period of 3 weeks. In contrast, after infection with DE16-H5N8B, 2 of 10 experimentally infected Pekin ducks as well as both sentinel ducks died. Increased virulence was mirrored and drastically emphasized using an intramuscular inoculation route in 1-week old Pekin ducklings, in which all DE16-H5N8B-infected animals succumbed within only 2 days. In addition, pathogenicity was also influenced by the duck species: Infection of juvenile Muscovy ducks confirmed the high virulence of the DE16-H5N8B virus, but this duck species revealed also a high susceptibility to the DE14-H5N8A virus. Previous works reported a higher virulence of many H5N1 gs/GD HPAIV for muscovy versus pekin type ducks, and showed that neurotropism/neurovirulence for muscovys, and younger age increased susceptibility of ducks to lethal and pathogenic effects of HPAIV infections^39–41^. Hence, we propose the intramuscular pathogenicity index using pekin ducklings as a feasible and easily standardizable test to categorize waterfowl pathogenicity in addition to the IVPI in chicken.

Overall, the differences in virulence for waterfowl together with the modulation by the age (e.g. high mortality in Pekin ducklings) and species of affected birds (Pekin ducks vs. Muscovy ducks) could explain the variable vulnerability and mortality of wild water birds observed in 2014/15 compared to 2016/17^12,42^. Interestingly, the observation of increased virulence of the H5N8 clade 2.3.4.4B over -A viruses resemble an earlier finding for HPAIV H5N1 clade 2.2, which reflected a further adaptation circle during endemic AIV circulation in waterfowl. According to Sturm-Ramirez and co-authors^43^, emerging HPAI H5N1 viruses of late 2002 and 2003 in Hong Kong revealed an increased virulence for ducks including the induction of brain lesions, confirming field observations during the Hong Kong outbreaks. In addition, an aggravating influence on virulence of one or more internal segments reassorted in the current isolate is likely and needs to be ascertained by follow-up studies.

In conclusion, as both H5N8 clades are derived from different ancestors^12^, this would suggest that duck pathogenicity evolved independently, presumably in Asia. It may be speculated that the epidemiological situation in Asia builds up a selection pressure that favors HPAIV H5 with enhanced duck pathogenicity. It remains unclear, however, whether this acquired biological function is the mere result of prolonged endemic circulation in (vaccinated) duck populations or whether this conveys an advantage when maintained in wild bird populations following spill-over infection from poultry.

Furthermore, the observed pathogenicity pattern obviously allowed for efficient transmission by migratory birds and subsequent spread into most European countries, including the incursion to hundreds of poultry farms with several million birds dying from infection or being culled^44^. The experimental data on virus shedding clearly demonstrate that both HPAI H5N8 clade 2.3.4.4A and B viruses efficiently replicated in ducks and were transmitted to co-housed syn- and allospecific birds. For both viruses, oro-pharyngeal shedding superseded cloacal excretion by a factor of up to 100 and lasted up to 7 dpi. High level oropharyngeal shedding indicates the importance of virus replication in the epithelium of the upper respiratory tract, including the infraorbital sinuses. This conclusion is in agreement with an observed strong antigen signal in the sinuses, but rare antigen detection in the intestine. However, this opposes the paradigm of preferential replication in the gastrointestinal tract of (low pathogenic) influenza viruses in aquatic birds^45,46^. Apparently, this reflects the differences in biological properties of the long circulating gs/GD-like HPAI H5 viruses^47,48^. Elevated oro-pharyngeal shedding had also been observed during previous experimental infections of ducks with clade 2.3.4.4 group A HPAIV H5Nx^18,19,31,34,38,49–52^.

The mechanisms underlying this shift of pathogenicity are currently not well understood and have to be studied in the future. Likely, beside the HA gene of DE16-H5N8B also internal genes contribute to the pathogenicity in ducks^53^. The presented intramuscular pathogenicity index in Pekin ducklings may serve in the future as a standardized test to study differences in virulence of HPAIV strains for waterfowl.

### Zoonotic potential—infection of mice, ferrets and human ex vivo  lung explant culture

Interestingly, the DE16-H5N8B virus efficiently replicated in mouse lungs without prior adaptation. A low infectious dose of 10^2^ TCID_50_/animal resulted in a mortality of 60%, while animals receiving higher dosages had to be euthanized before 10 dpi. The final calculated mean lethal dose 50 for the Balb/c mouse experiments was 2.4 × 10^2^/animal, demonstrating clearly a more virulent phenotype than reported previously for H5N8A viruses by others^18,54^. Whether this variability reflects different experimental designs e.g., age of the mice, endpoint criteria or represents different molecular determinants of influenza virus pathogenesis in mice will be part of future studies. In addition, substantial virus titers in lungs and systemic spread of virus to the brain were verified. Therefore, DE16-H5N8B replicated in mice and demonstrated a virulent phenotype. This phenotype seems to be correlated with the high pathogenicity in Pekin ducks, a hypothesis that has to be further evaluated in future studies, e.g., using reassortants of both groups of HPAIV H5N8 or testing further HPAIV strains with a difference in duck pathogenicity.

On the contrary, low virulence was observed for DE16-H5N8B in the ferret model. The clinical signs were mild and only minor changes in both body weight and body temperature were detected. Virus was re-isolated from a single nasal wash sample only, and moderate viral titers were determined from few organ samples. Positive antigen staining in tonsil samples substantiate the replicative potential of HPAIV H5 viruses detected by Lipatov and colleagues^55^. Furthermore, direct contact ferrets did not seroconvert, thereby confirming that DE16-H5N8B was not transmitted between ferrets co-housed in the same cage. HA, NA and PB2 Sanger sequences of the recovered virus isolate after one ferret passage exhibited 100% nucleotide identity to the inoculated virus (data not shown). Overall, these results are consistent with previous pathogenicity studies with H5N8 clade 2.3.4.4A viruses isolated from South Korea also exhibiting a low virulence in the ferret model after intranasal inoculation^24^. Despite the fact that DE16-H5N8B was highly virulent for Balb/C-mice, the outcomes of the ferret experiments support the conclusion for a very low zoonotic risk of these viruses.

To provide more data for a profound risk assessment, we also evaluated DE16-H5N8B in a human lung explant model, in which viruses known to cause disease in humans replicate efficiently^56,57^. Interestingly, the human tissue did not support DE16-H5N8B propagation unlike the human H3N2 and zoonotic H5N1 control strains which is fully consistent with the low infectivity of DE16-H5N8B in ferrets, and suggests a low potential for zoonotic transmissions. The currently available data suggest a lack of species adaptation of this virus to human lung cells, which will need to be examined in follow-up studies: For instance, the H5N8 virus induced more antiviral IFN-beta in human lung cultures than the productively replicating control strains thereby impacting on virus growth by innate immune stimulation.

Overall, high virulence and transmissibility in waterfowl are biological key features of the examined recent HPAIV H5N8 strain of clade 2.3.4.4 B, and judging on the massive mortality in wild birds in Europe, also of further reassortants of this clade. Interestingly, the augmented virulence for Pekin ducks seems to be connected with a higher virulence in Balb/C mice. Thus, the data from the Balb/C mouse model may be predictive for the virulence of HPAIV H5-viruses in ducks. The results obtained by the ferret infection model were fully consistent with findings in a human lung explant infection model. The summarizing conclusion of a low zoonotic potential of these viruses is further supported by the observation that up to now no human cases have been reported for the H5N8B strain, despite the very broad distribution in the wild bird and poultry populations in many different countries and the re-emergence of the same strain in the last months in several European countries.

Overall, the comprehensive study reported here provides important proof-of-concept data useful for future risk assessments of HPAIV strains, and we suggest to use the ferret infection model together with the human lung explant culture model for the routine evaluation of all new HPAIV strains.

## Material and methods

### Ethics statement

The animal experiments were evaluated by the ethics committee of the State Office of Agriculture, Food safety, and Fishery in Mecklenburg – Western Pomerania (LALLF M-V) and gained legal governmental approval under the registration numbers LVL MV/TSD/7221.3-1.1-023/13 and 7221.3-2.2-002/09. All procedures were carried out in approved biosafety level 3 facilities. Use of tumor-free normal human lung tissue was approved by the ethics committee at the Charité clinic (projects EA2/050/08 and EA2/023/07) and written informed consent was obtained from all patients.

### Viruses and virus propagation

HPAIV H5N8 of clade 2.3.4.4 group A, namely A/turkey/Germany-MV/R2472/2014 (DE14-H5N8A) and 2.3.4.4 group B A/tufted_duck/Germany/AR8444-L01987/2016 (H5N8) (DE16-H5N8B), were obtained from the Friedrich-Loeffler-Institut (FLI) virus repository. Viruses were propagated in the allantoic cavity of 11-day-old embryonated eggs from specific pathogen free (SPF) chicken, and, after clarification of the harvested allantoic fluids by centrifugation, stored at −70 °C until further use. Viral titers of the avian viruses were determined by virus titration with Madin-Darby canine kidney (MDCK) cells. The genome sequences of DE14-H5N8A and DE16-H5N8B are available from the EpiFlu database of the global initiative on sharing all influenza data (GISAID) (Accession numbers EPI544756, EPI544759, EPI548426 – EPI548431 and EPI860390 – EPI860397).

As reference virus stocks of the human influenza A/Panama/2007/1999 (Pan H3N2) and A/Thailand/1 (Kan-1)/2004 (Thai H5N1, clade 1) viruses were propagated in MDCK type II cells for two days at 37 °C as described previously^58^. Virus supernatant was collected, cleared of debris and stored at −80 °C. Titers of the viruses used in human lung infection were determined by standard plaque titration assay.

### Experimental animal infection

#### Birds (group assignment details see table S2)

(A) Intravenous pathogenicity index (IVPI) testing in chickens was done according to standard procedures^59^. (B) Intramuscular pathogenicity index (IMPI) testing in 1-week old Pekin- (*Anas platyrhynchos var. domesticus*) and Muscovy ducklings (*Cairina moschata*) followed the principles of the IVPI in chicken, but with injection of virus into the caudal femoral muscles and subsequent clinical scoring for 10 days. Pekin ducks were selected as synspecific equivalents to mallards^60^. In contrast, Muscovy ducks are derived from South American ducks but are also commonly used commercially in Europe. As duck species differ in their genetics and susceptibility for avian influenza viruses, ducklings from both species were inoculated with either DE14-H5N8A or DE16-H5N8B. (C) Oculo-nasal (o.n.) infection with DE14-H5N8A was done in co-housed adult Pekin ducks and geese of the Pommeranian land race (10 birds/group), whereas o.n. infection with DE16-H5N8B focused on adult Pekin ducks only, each time applying 10^6^ TCID_50_. On day one after infection, 2 birds from the same waterfowl species as well as adult SPF-chicken were housed together with the inoculated waterfowl and served as sentinels. Housing and handling of birds were done as described previously^61,62^.

#### Mammalian models

Mouse lethal dose 50 (MLD_50_) was determined in 4-week-old BALB/c mice (group assignment see table S3) inoculated intranasally (i.n.).

Eight 3- to 6-month-old healthy and influenza-negative ferrets (*Mustela putorius furo*) were inoculated i.n. with 10^6^ TCID_50_ of DE16-H5N8B in a total volume of 50 µL and housed together with four ferrets that served as transmission sentinels by direct contact (in a 1:1 setting). For details on monitoring, sampling and testing of animals see supplemental materials.

### Infection of human lung tissue ex vivo

The infection experiments were done as described previously^57,63,64^ and the lung tissues were tested for infectious virus at indicated times post infection. For details see supplemental material.

## Electronic supplementary material

Supplemental Material
