# Supplementary material for: A novel European H5N8 influenza A virus has increased virulence in ducks but low zoonotic potential
Source: Emerg Microbes Infect. 2018 Jul 19;7:132. doi: 10.1038/s41426-018-0130-1 (PMC6053424; doi:10.1038/s41426-018-0130-1)

# Supplemental information

## Supplemental material and methods

*Experimental infection of ducks, geese and chicken with DE14-H5N8A and DE16-H5N8B*

White leghorn chicken (*Gallus gallus var. domesticus*) were hatched and raised at the FLI from SPF-eggs purchased from VALO BioMedia, Germany. One day old Pekin ducklings (*Anas platyrhynchos var. domesticus*) and Muscovy ducklings (*Cairina moschata*) were obtained from commercial sources. Adult 6-7 month old throughbreed ducks (Anas platyrhynchos var. domesticus, “Rouen Claire”) and geese (*Anser anser var. domesticus,* ”Pommeranian goose”) were obtained from a local breeder and tested AIV-seronegative by ELISA (ID-Vet, France) before inoculation. Animal numbers and group assignment are summarized in table S2. Intravenous pathogenicity index testing (IVPI) was done according to standard procedures [1]. Intramuscular pathogenicity index (IMPI) testing in one week old Pekin and Muscovy ducklings followed the procedure of the IVPI in chicken, but with injection of virus into the caudal femoral muscles and subsequent clinical scoring for 10 days. In two subsequent experiments aadult ducks were inoculated oculo-nasally (o.n.) with $10^6$ $TCID_{50}$ of either DE14-H5N8A or DE16-H5N8B. During the first infection experiment geese (n=10) were simultaneously infected with DE14-H5N8A and housed together with inoculated ducks. Virus replication was monitored by pharyngeal- and cloacal-swabs on day 2, 4, 7, 10 and 14 and on day 2 and 4 2 animals were sacrificed to investigate birds pathologically. During the second trial with DE16-H5N8B, monitoring was intensified during acute phase with taking swab samples daily form day 1-7 p.i. and subsequently on day 10 and 16 p.i.On day one after infection 2 animals from the same waterfowl species as well as adult SPF-chicken (n=4 for DE14-H5N8A and n=10 for DE16-H5N8B) were housed together with the

inoculated waterfowl and served as sentinels. Animals were monitored daily for clinical signs for a period of up to 21 days. Animals the suffered severe disease like pronounced lethargy or apathy were sacrificed before and considered dead on the subsequent day. The intramuscular pathogenicity and clinical scoring indices (IMPI) were calculated based on the following parameters: healthy (0), ill (1) (exhibiting one of the following clinical signs: somnolence, labored breathing, cyanosis, neurological symptoms), (2) severely sick (showing at least two of the previous clinical signs) or (3) dead. Oropharyngeal and cloacal swabs were taken at indicated days post infection (dpi) or as combined oropharyngeal/cloacal swabs prior to infection and stored in 1 ml medium (mixture of equal volumes of Eagle MEM (Hanks' balanced salts solution), 2mM L-Gln, nonessential amino acids, adjusted to 850 mg/L NaHCO3, 120 mg/L sodium pyruvate, 10% FBS, pH 7.2). All animals that died during the o.n. inoculation experiments were subjected to pathological investigation and subsequent sampling of organs for virological and histological analyses. In case of waterfowl inoculated with DE14-H5N8A, with no spontaneous deaths, two randomly selected animals from each species were euthanized on day 2 and 4 for investigations.

*Experimental infection of mice and ferrets with DE16-H5N8B*

**Mice**: To determine the mouse lethal dose 50 ($MLD_{50}$), based on $TCID_{50}$/mL of DE-H5N8B, a total of 52 4 week-old BALB/c mice were used. Table S3 summarizes mice numbers per dosage groups used. Mice were anesthetized by inhalation of 5 % isoflurane and inoculated intranasally (i.n.) with 30 µL 10-fold serial dilutions from $10^2$ to $10^6$ $TCID_{50}$/animal. Mice serving as control animals were manipulated the same way as inoculated animals, without receiving virus inoculum. The $MLD_{50}$ was calculated as log10 $TCID_{50}$/mL. All $TCID_{50}$ and $MLD_{50}$ calculations were performed using the method of Spearman and Kärber [2,3]. Mice were monitored daily for weight loss, clinical signs and mortality for 14 days. The percent weight loss was calculated from the original starting

weight. Mice showing more than 25% of body weight loss (compared to body weight mean values at the specific age, provided by the supplier of animals) were considered to have reached the experimental end point and were euthanized. To determine virus titres in various tissues, three mice of each group were euthanized on 3 dpi, in addition three mice from the groups $10^3$, $10^5$, $10^6$ were euthanized on 6 dpi, and organs (i.e., lungs, conchae, heart, and brains) were harvested for histopathological- and virological examinations.

**Ferrets**: 3-6 month-old healthy and influenza-negative ferrets (*Mustela putorius furo*) were reared at the FLI and housed in cages during the experiment. Cages were separated by stainless-steel grids to prevent direct contact. Eight ferrets were anesthetized by inhalation of 5 % isoflurane and infected intranasally with $10^6$ $TCID_{50}$ of the DE16-H5N8B virus in a total volume of 50 µL. Four ferrets served as transmission sentinels by direct contact (in a 1:1 setting). Animals were monitored daily for clinical signs (nasal discharge, labored breathing, reduced activity, fever, body weight loss, or neurological symptoms); they were weighed every second day, and body temperatures were recorded by the use of subcutaneous implantable temperature-logging devices (Plexx, Elst, The Netherlands). Blood samples were drawn from the Vena saphena under light anaesthesia with isoflurane prior to infection or on the day of euthanasia, respectively. Nasal washes were collected every other day from all ferrets to measure virus replication by applying 1 ml phosphate-buffered saline (PBS) into each nostril. From genome positive nasal washing samples virus titration attempts were performed using MDCK cell cultures. Four inoculated animals were euthanized on 3 dpi, dissected and spleens, tracheas, lungs (divided into left and right parts), conchae, cerebellum, and cerebrum were removed and stored at -70 °C until further use.

*Organ homogenization*

Tissue samples of individual animals were re-suspended in 1 mL of medium (mixture of equal volumes of Eagle MEM (Hanks' balanced salts solution) and Eagle MEM (Earle's

balanced salts solution), 2mM L-Glutamine, nonessential amino acids, adjusted to 850 mg/L $NaHCO_3$, 120 mg/L sodium pyruvate, 10% fetal bovine serum (FBS), pH 7.2) supplemented with 10 % FBS plus antibiotics (1% penicillin-streptomycin). A single stainless steel bead (5 mm) per organ sample was added and samples were homogenized in a 2 ml collection tube for 2 min in a TissueLyser (Qiagen, Hilden, Germany).

*Quantitative Real-time RT-PCR*

Viral RNA was extracted from 200 µl of the supernatant of organ homogenates or swab samples using the MagAttract® Virus Mini M48 kit (Qiagen, Hilden, Germany) according to the manufacturer´s instructions on a Biosprint 96 platform (Qiagen).

Virus quantification was performed as cell culture titration and/or based on quantitative real-time reverse transcriptase PCR (RT-qPCR) of virus genome. For ferret and mouse experiment samples, an assay based on viral genome segment 2 (PB1) was established. The primer and probe set of the generic PB1 assay (summarized in table S4) was used to determine the quantification cycle (Cq) in swabs, nasal washes, and organs using the one-step RT-qPCR Kit qScript™ XLT One-Step RT-qPCR ToughMix® (Quantabio, Beverly USA). The RT-qPCR assay was optimized for using a total volume of 12.5 µl. Briefly, for a single well 1.75 µl RNase-free water, 6.25 µl 2x RT-PCR buffer, and 1.0 µl primer-probe-mix for template detection as well as 1.0 µl primer-probe-mix for detection of the internal extraction control RNA, were pooled as a master mix. Finally, 2.5 µl RNA template was added and the reaction was run on an CFX96 machine. Genome copy numbers per µl were determined by absolute quantification via droplet PCR technique and log10 dilution standards were used within the subsequent RT-qPCR assay. The generated standard curve exhibited the following characterizing values: $y = -3.519x + 36.474$; $R2 = 0.999$.

Avian sample analysis was performed using a segment 7 (M) based RT-qPCR as previously described [4]. A standard curve for virus quantification was generated using

extracted viral RNA from diluted HPAIV H5N8 suspensions with known infectivity titer by RT-qPCR targeting the M gene. To relate M-specific Cq values to viral infectivity in the examined sample, Cq values from these extracts were plotted on the generated standard curve linking infectivity with Cq values.

*Histopathology and immunohistochemistry*

Tissues were sampled in 4 % neutral buffered formaldehyde, processed and embedded in paraffin wax using a Leica ASP 300S fully enclosed tissue processor (Leica Biosystems, Nussloch, Germany), sectioned at 2-4 µm thickness using a Hyrax M55 electronic rotary microtome (Carl Zeiss Microimaging GmbH, Jena, Germany), mounted on Superfrost plus glass slides (Menzel Gläser, Braunschweig, Germany), stained with hematoxylin and eosin using a Medite TST 44.000C automatic tissue stainer (Medite, Burgdorf, Germany; Romeis Mikroskopische Technik. Editors: Mulisch Maria, Welsch Ulrich. 18th Edition 2010 Spektrum Akademischer Verlag, Heidelberg), and screened for histopathological changes using an Axio Imager M2 microscope equipped with 10x, 20x, and 40x Plan Neofluar objectives, and a AxioCam ICc3 3.3 megapixel digital camera (Carl Zeiss Microscopy GmbH, Jena, Germany). The tissues of ducks and chicken were semi-quantitatively assessed for necrosis and / or necrotizing inflammation as well as lymphatic apoptosis, necrosis and / or depletion on a 0 to 3 severity scale: 0 = no obvious lesion; 1 = mild, 2 = moderate, 3 = severe lesion(s).

Immunohistochemistry was performed on serial sections to detect influenza A virus-antigen using the avidin-biotin-peroxidase-complex method (Vectastain PK 6100, Vector, Burlingame, CA, USA) with citric buffer (10mM, pH 6,0) pre-treatment, a polyclonal rabbit anti- influenza A FPV/Rostock/34-virus-nucleoprotein antiserum (diluted 1:750) [5], 3-amino-9-ethyl-carbazol as chromogen (Agilent Technologies, Santa Clara, CA USA) and hematoxylin counterstain. The specificity of the immunohistochemical reaction was

confirmed by the use of negative tissues from mock-infected mice from this experiment, negative tissues from an archival of diagnostic cases of non-infected chicken, of a non-infected ferret (P17-348), as well as validated positive avian tissues from the recent H5N8 outbreak [6]. As negative controls, serial sections were treated with rabbit serum instead of the primary polyclonal antiserum. The distribution of influenza A virus nucleoprotein was semi-quantitatively assessed for each organ by scoring on a 0 to 3 severity scale: 0 = negative; 1= focal or oligofocal, 2= multifocal, 3= coalescing to diffuse immune-reactive nuclei. This grading was subdivided for endothelial cells and parenchymal cells, respectively for chicken and ducks.

*Serology*

Serum samples from animals were heat-inactivated at 56 °C for 30 min and analyzed by means of a commercial enzyme-linked immunosorbent assay (ELISA) for the presence of antibodies against IAV nucleoprotein (NP) (ID Screen® Influenza A Antibody Competition ELISA kit, ID-vet, Montpellier, France) and subtype H5 (ID Screen® Influenza H5 Antibody Competition ELISA kit, ID-vet, Montpellier, France) according to the manufacturer´s instruction. HI assays against antigen of DE-R2472/2014A, and DE16-H5N8B were performed according to standard protocols [1].

*Infection of human lung tissue ex vivo.*

Tumor-free normal human lung tissue was cut into small pieces (weight approx. 0,1 – 0,2 mg per piece) and incubated in RPMI 1640 medium overnight to wash off clinically applied antibiotics. Use of human explant tissue was approved by the ethics committee at the Charité clinic (projects EA2/050/08 and EA2/023/07) and written informed consent was obtained from all patients. The infection experiments were done in RPMI 1640 medium supplemented with 0.3 % bovine serum albumin, 2 mM L-glutamine and 25mM HEPES at 37

°C with 5 % $CO_2$ as described previously [7-9]. Human lung tissue explants were inoculated with 2 × $10^5$ PFU for replication analyses or 1 × $10^6$ PFU for ELISA and immunofluorescence studies of influenza viruses Pan H3N2, Thai H5N1 or DE16-H5N8B for 1.5 h. Excess virus was removed by washing with phosphate-buffered saline (PBS) and lung tissue was incubated for up to 48 hpi (hours post infection). For each experiment, tumor-free tissue specimens from at least three donors were analyzed. For replication analysis, at 1, 16, 24 and 48 hpi supernatants of infected lung tissue were harvested and viral titers were determined by standard plaque titration assay. For immunofluorescence and ELISA analyses, samples were prepared at 24 hpi.

*Enyzme-Linked Immunosorbent Assay (ELISA).*

Supernatants of infected human lung tissue were collected 24 hpi, cleared of debris and stored at -80 °C. IL1β and IFNβ levels were measured according to the manufacturer's instructions with the "Human IL-1 beta/IL-1F2 DuoSet ELISA" (R&D Systems) or the "Human IFN-beta DuoSet ELISA" (R&D Systems), respectively.

*Confocal microscopy*

Human lung tissue was infected with human Pan H3N2, Thai H5N1 or avian DE16-H5N8B and cultured for 24 h. Tissue samples were fixed and routinely paraffin embedded as described before [3]. After deparaffinization, slices were immunostained with fluorescently labeled Alexa Fluor 488 influenza A virion-specific antibody (Serotec, Puchheim, Germany) to detect virus-infected cells (green channel) and pro-SP-C (Chemicon, AB3786; red channel) to identify type II pneumocytes. An Alexa Fluor 594-labeled anti-rabbit F(ab′)2 fragment was applied as the secondary antibody. Nuclei were counterstained with DAPI (4′,6-diamidino-2-phenylindole). Tissue sections were mounted in Mowiol and analyzed by spectral confocal microscopy using a 780 laser-scanning microscope (objectives 63x, zoom: 1.9; Plan-Neofluor/oil, NA 1.3, Plan-Apochromat/oil, NA 1.4; Zeiss).

*Statistical analysis*

Comparison of clinical scores was done initially by Kruskal-Wallis-Test to test for principal differences between groups and subsequently (post-hoc) Wilcoxon-Mann-Whitney tests for pairwise comparison using the Benjamini-Hochberg procedure. Data for replication analysis were presented as mean and standard error of the mean of at least three donors within independent experiments. Data for cytokine ELISA were presented as mean with standard deviation. Using GraphPad Prism 7 software, for replication analysis the Mann-Whitney U test was performed to delineate significant differences between data points. For cytokine ELISAs, statistical analysis was conducted by applying One-Way-Anova test and subsequently Dunn's multiple comparisons test using GraphPad Prism 7 software. For all assays, a p value $\leq 0.05$ was considered significant, with $p \leq 0.05$ depicted as (*), $p \leq 0.01$ shown as (**) and $p \leq 0.0001$ shown as (****).

## Supplemental figures legends

**Supplemental figure S1: Viral shedding of Geese after HPAIV DE14-H5N8A infection**

Viral sheeding was monitored by RT-qPCR from swabs taken from oro-pharynx (A) or cloaca (B) from Geese infected oculo nasal with DE14-H5N8A. Individual results of detected RNA copy numbers are given as virus equivalents (VE), calculated by using a set of standards applied in each run. All RT-PCR negative samples are depicted as 10 VE, i.e. under the detection limit. Individual inoculated animals (○) and sentinel animals (▼) as well as arithmetic mean and standard deviation of positive swab samples from inoculated birds (red dot) are shown. Numbers within the figures give number of virus positive -and number of total tested infected animals for each time point.

**Supplemental figure S2: Sero-reactivity of HP AIV H5N8 infected birds.**

Sera from Geese (A) or ducks (B) infected with DE14-H5N8A or ducks infected with DE16-H5N8B (C) were taken at indicated times after infection and tested by HI, using the homologous antigen.

**Supplemental figure S3: Viral load in organs of HPAIV H5N8 infected birds.**

Organs of ducks (D) and geese (G) infected with DE14-H5N8A were sampled from 2 randomly selected animals on day 2 (d2) or day 4 (d4) after infection or from chickens that died on day 4 and 5 post contact respectively (A). Organ viral load after DE16-H5N8B infection was tested at the time of spontaneous dead for indicated times after infection for ducks (B) and chicken (C). Individual results of detected RNA copy numbers are given as virus equivalents (VE), calculated by using a set of standards applied in each run. Blue line indicating the cut of value.

**Supplemental figure S4: Characteristic light microscopic findings in chicken infected with DE16-H5N8B.**

(A) Chicken, 3 d.p.i., brain. Moderate, subacute necrotizing polioencephalitis with infiltration of phagocytic microglia (arrow). (B) Chicken, 3 dpi., brain. Multiple foci (arrow) of influenza A nucleoprotein-immunoreactive neurons and glial cells. (C) Chicken, 3 dpi., liver. Mild, acute, hepatocellular necrosis (necrotizing hepatitis) characterized by small nests of shrunken hepatocytes with pyknotic and karyorrhectic nuclei (arrow) and few infiltratin heterophils. (D) Chicken, 3 dpi., liver. The influenza A virus-nucleoprotein-immunoreactivity displays a multifocal perivascular (star) and perisinusoidal pattern suggestive of endothelial and / or Kupffer cells and only scant, focal immunoreactive hepatocytes. (E) Chicken, 3 dpi., pancreas. Mild, acute, pancreatic necrosis (necrotizing pancreatitis) characterized by small nests of either swollen and ruptured or shrunken hypereosinophilic exocrine pancreatocytes with pyknotic and karyorrhectic nuclei (arrow). (F) Chicken, 3 dpi., pancreas. Multiple foci (star) of influenza A virus-nucleoprotein-immunoreactive exocrine pancreatocytes. (G) Chicken, 7 dpi., spleen. Severe, acute, lymphatic apoptosis and necrosis characterized by cytoplasmic hypereosinophilia, nuclear pyknosis and karyorrhexis (arrow) within a follicular area (star) of the white pulp. (H) Chicken, 7 dpi., spleen. Especially the follicles (star) of the white pulp are surrounded by a rim of influenza A virus-nucleoprotein-immunoreactive cells interpreted as follicular dendritic cells and macrophages. Furthermore there is faintly immunoreactive debris within the follicles and oligofocal cells interpreted as macrophages / dendritic cells and endothelial cells within both white and red pulp. A, C, E, G Hematoxylin eosin, bar = 20µm. B, D, F, G Influenza A virus-nucleoprotein immunohistochemistry, avidin-biotin-peroxidase complex method with 3-amino-9-ethyl-carbazol as chromogen and hematoxylin counterstain, bar = 50 µm.

## Supplemental tables

**Supplemental table S1: Histopathological lesions and DE16-H5N8B tissue tropism in mice and ferrets.**

| | Mouse (DE16-H5N8B) | Mouse (control) | Ferrets (DE16-H5N8B) | Ferrets (archival control) |
|---|---|---|---|---|
| Brain. encephalitis[1] | 4/5 (80%)[3] | 0/3 (0%) | 0/4 (0%) | 0/1 (0%) |
| Brain. influenza A virus-nucleoprotein[2] | 3/5 (60%) | 0/3 (0%) | 0/4 (0%) | 0/1 (0%) |
| Heart. myocarditis[1] | 1/5 (20 %) | 0/3 (0%) | 0/3 (0%) | 0/1 (0%) |
| Heart. influenza A virus-nucleoprotein[2] | 1/5 (20 %) | 0/3 (0%) | 0/3 (0%) | 0/1 (0%) |
| Lung. pneumonia[1] | 3/5 (60%) | 0/3 (0%) | 0/4 (0%) | 0/1 (0%) |
| Lung. influenza A virus-nucleoprotein[2] | 0/5 (0%) | 0/3 (0%) | 0/4 (0%) | 0/1 (0%) |
| Nasal cavity. rhinitis[1] | n.d. | n.d. | 2/4 (50%) | 0/1 (0%) |
| Nasal cavity. influenza A virus-nucleoprotein[2] | n.d. | n.d. | 0/4 (0%) | 0/1 (0%) |
| Tonsilla palatina. influenza A virus-nucleoprotein[2] | n.d. | n.d. | 1/2 (50 %) | 0/1 (0%) |

[1] determined by histopathology

[2] determined by immunohistochemistry

[3] number of positive animals / number of total animals (positive animals %); n.d. = not done

**Supplemental table 2: Animal numbers used within the inoculation experiment of poultry with DE-H5N8A / DE-H5N8B**

| | adult | | | | | | duckling (IMPI) | |
|---|---|---|---|---|---|---|---|---|
| | peking duck inoculated | sentinel birds in direct contact to inoculated peking ducks | | geese | sentinel birds in direct contact to inoculated geese | | peking duck | muscovy duck |
| | | ducks | chicken | | geese | chicken | | |
| DE-H5N8A | 10 | 2 | 4 | 10 | 2 | 4 | 10 | 10 |
| DE-H5N8B | 10 | 2 | 10 | - | - | - | 10 | 10 |

**Supplemental table 3: Animal numbers used within the inoculation experiment of mice with DE-H5N8B**

| | dosage group | | | | | |
|---|---|---|---|---|---|---|
| | $10^2$ | $10^3$ | $10^4$ | $10^5$ | $10^6$ | Control |
| $MLD_{50}$ | 5 | 5 | 5 | 5 | 5 | 3 |
| 3 dpi* | 3 | 3 | 3 | 3 | 3 | - |
| 6 dpi# | - | 3 | - | 3 | 3 | - |

* intended to be euthanized three days post inoculation

# intended to be euthanized six days post inoculation

**Supplemental table 4: Primers and probes used for a generic detection of influenza A virus-specific RNA in diagnostic materials from mice and ferrets in this study.**

| Designation | Sequence 5`⇒ 3` | Concentration of primer and probes in the primer-probe-mix |
|---|---|---|
| ***Pan-IAV assay*** | | |
| **IAV-PB1** | **(this study)** | |
| IAV-PB1_120F | CATTTGAATGGAYGTCAAYCCGA | 20 µM |
| IAV-PB1_271R | CTGTTDACYGTGTCCATDGTGTA | 20 µM |
| IAV-PB1_247as_FAM | FAM CCWGTYCCYGTYCCATGGCTGTA-BHQ1 | 5 µM |
| ***internal extraction control assay*** | | |
| **β-Actin Mix-HEX** | **(modified from [10]** | |
| ACT2-1030-F | AGCGCAAGTACTCCGTGTG | 5 µM |
| ACT-1135-R | CGGACTCATCGTACTCCTGCTT | 5 µM |
| ACT-1081-HEX | HEX-TCGCTGTCCACCTTCCAGCAGATGT-BHQ1 | 2.5 µM |

## Supplemental references


1 Commission, E. 2006/437/EC: Commission Decision of 4 August 2006 approving a Diagnostic Manual for avian influenza as provided for in Council Directive 2005/94/EC. Report No. ISSN 1725-2555, 16 (2006).

2 Spearman, C. The Method of "Right and Wrong Cases" (Constant Stimuli) without Gauss's Formula. *Br J Psychol* **2**, 227-242, doi:10.1037/h0063767 (1908).

3 Kärber, G. Beitrag zur kollektiven Behandlung pharmakologischer Reihenversuche. *Archiv f experiment Pathol u Pharmakol* **162**, 480-483, doi:10.1007/BF01863914 (1931).

4 Hoffmann, B. *et al.* New real-time reverse transcriptase polymerase chain reactions facilitate detection and differentiation of novel A/H1N1 influenza virus in porcine and human samples. *Berl Munch Tierarztl Wochenschr* **123**, 286-292 (2010).

5 Klopfleisch, R., Werner, O., Mundt, E., Harder, T. & Teifke, J. P. Neurotropism of highly pathogenic avian influenza virus A/chicken/Indonesia/2003 (H5N1) in experimentally infected pigeons (Columbia livia f. domestica). *Vet Pathol* **43**, 463-470, doi:10.1354/vp.43-4-463 (2006).

6 Pohlmann, A. *et al.* Outbreaks among Wild Birds and Domestic Poultry Caused by Reassorted Influenza A(H5N8) Clade 2.3.4.4 Viruses, Germany, 2016. *Emerg Infect Dis* **23**, 633-636, doi:10.3201/eid2304.161949 (2017).

7 Hocke, A. C. *et al.* Emerging human middle East respiratory syndrome coronavirus causes widespread infection and alveolar damage in human lungs. *Am J Respir Crit Care Med* **188**, 882-886, doi:10.1164/rccm.201305-0954LE (2013).

8 Weinheimer, V. K. *et al.* Influenza A viruses target type II pneumocytes in the human lung. *J Infect Dis* **206**, 1685-1694, doi:10.1093/infdis/jis455 (2012).

9 Berg, J. *et al.* Tyk2 as a target for immune regulation in human viral/bacterial pneumonia. *Eur Respir J* **50**, doi:10.1183/13993003.01953-2016 (2017).

10 Toussaint, J. F. *et al.* Bluetongue in Belgium, 2006. *Emerg Infect Dis* **13**, 614-616, doi:10.3201/eid1304.061136 (2007).

**Figure S1**

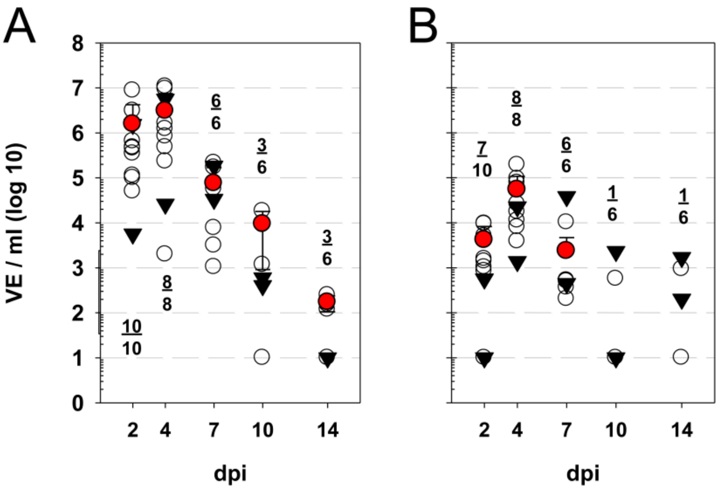

Figure S2

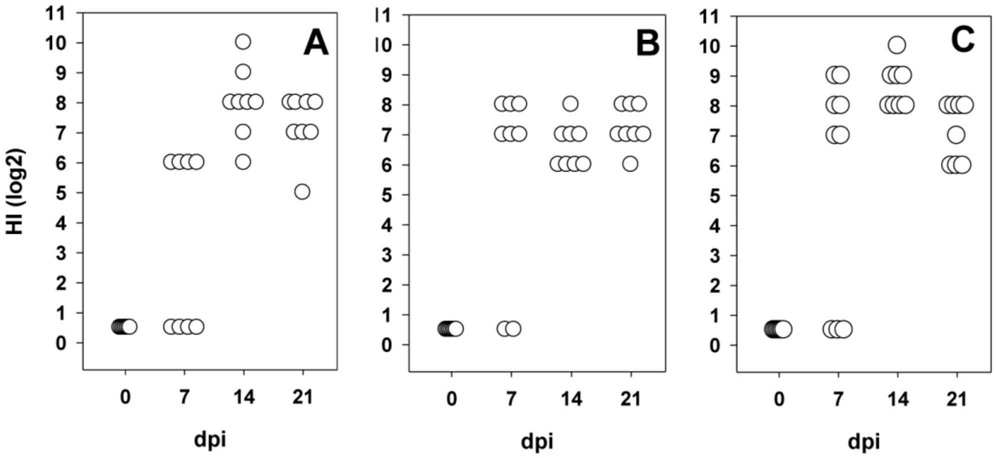

Figure S3

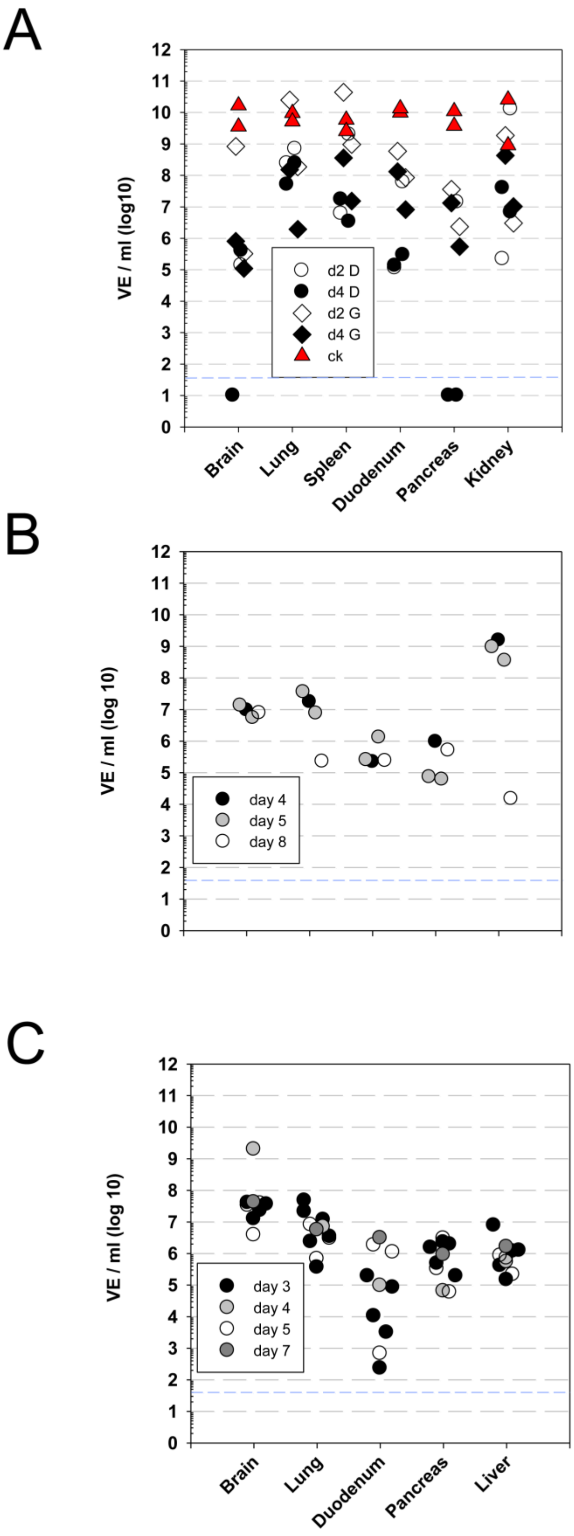
A
VE / ml (log10)
d2 D
d4 D
d2 G
d4 G
ck
Brain
Lung
Spleen
Duodenum
Pancreas
Kidney
B
VE / ml (log 10)
day 4
day 5
day 8
C
VE / ml (log 10)
day 3
day 4
day 5
day 7
Brain
Lung
Duodenum
Pancreas
Liver

Figure S4:

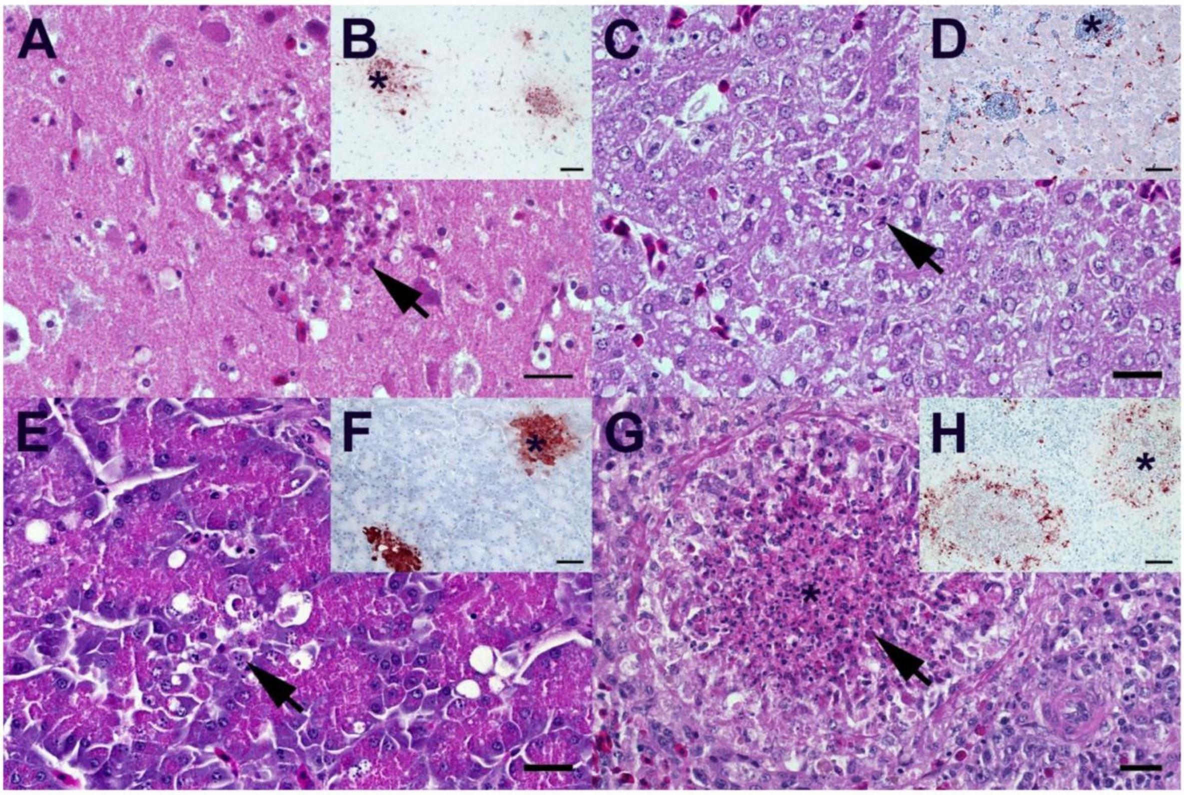

Supplement: Supplementary file 1 — Supplemental Material [file 41426_2018_130_MOESM1_ESM.pdf]
